# Sensitivity of a point of care tick-test for the development of Lyme borreliosis

**DOI:** 10.1186/1756-3305-6-338

**Published:** 2013-12-04

**Authors:** Hein Sprong, Arieke Docters van Leeuwen, Manoj Fonville, Margriet Harms, Arnold J van Vliet, Wilfrid van Pelt, José A Ferreira, Cees C van den Wijngaard

**Affiliations:** 1 Centre for Infectious Disease Control Netherlands, National Institute for Public Health and Environment, Antonie van Leeuwenhoeklaan 9 P.O. Box 1 3720 BA, Bilthoven, The Netherlands; 2 Environmental Systems Analysis Group, Wageningen University, Wageningen, The Netherlands

**Keywords:** Lyme borreliosis, Self-test, *Ixodes ricinus*, Validation, Post-exposure prophylaxis

## Abstract

**Background:**

A commercially available self-test for the detection of *Borrelia burgdorferi* sensu lato in ticks was evaluated for its ability to predict erythema migrans formation.

**Findings:**

The self-test was performed on 127 *Ixodes ricinus* from 122 humans that reported tick bites at enrolment and occurrence of symptoms during follow-up. The self-test gave negative results on all the 122 individuals, 14 of whom reported erythema migrans (EM) at follow-up of which 10 were confirmed by their GP. The estimated sensitivity of the self-test for prediction of EM formation is 0% (95% CI: 0%-28%).

**Conclusions:**

This self-test is not suitable for reducing the number needed to treat in a post-exposure prophylaxis setting as it already missed all the obvious early Lyme borreliosis cases.

## Findings

Lyme borreliosis is an emerging tick-borne disease, caused by spirochetes of the *Borrelia burgdorferi* sensu lato species complex [1,2]. The most characteristic manifestation of early Lyme borreliosis is erythema migrans (EM), an expanding skin rash occurring after several days or weeks at the site of the tick bite [3]. Late and more serious Lyme borreliosis can manifest itself as a multi-system disease with several skin, neurological, cardiac and musculoskeletal manifestations [4]. Antibiotic treatment of EM commonly prevents the development of late and more severe disease stages. However, estimates indicate that around 25% of Lyme borreliosis patients do not detect or do not receive treatment for EM [5,6]. Prevention of Lyme borreliosis is difficult as no absolute control measures are available as yet [7,8]. Post-exposure prophylaxis after tick-attachment may be an effective way to reduce morbidity of Lyme borreliosis. In one randomized trial performed in the United States, prophylaxis was shown to prevent early Lyme borreliosis after a tick bite, with a number needed to treat (NNT) of 51 [9]. The NNT can be reduced if the ability of a test to predict the development of Lyme borreliosis is increased. Such a test could be based on Borrelia-infection and attachment time of the tick [10,11]. It requires a high sensitivity to be able to treat all persons at risk for Lyme borreliosis, and moderate specificity to be able to reduce the NNT. A self-test for the detection of *B. burgdorferi* sensu lato in ticks, the Careplus Tick-Test (Tropicare, Almere, Netherlands), is commercially available in Europe. This Careplus Tick-Test is based on the detection of *B. burgdorferi* sensu lato antigens in tick lysates. We evaluated the ability of this test to predict EM formation.

In an ongoing web-based national survey (http://www.tekenradar.nl), which started in 2012, more than 3000 individuals have reported tick bites, sent in the tick that bit them, and were asked to fill out online questionnaires, one at enrolment and follow-up questionnaires after three, six and nine months. EM formation was actively self-reported on the website or in the periodic questionnaires. For the test evaluation, we selected 122 individuals together with 127 *Ixodes ricinus* nymphs or adults, as 5 individuals sent in two ticks. To be able to assess the ability of the Careplus Tick-test to predict EM formation, we selected ticks from 25 individuals who reported EM, 45 individuals who did not report EM at follow up, and 52 individuals with unknown outcome by loss to follow-up at 3 months. This is not a random sample from all reported tick bites as, we selected a relatively large number of ticks from individuals with reported EM. In order to consider only ticks that caused EM, we have further restricted the EM cases by requiring that: 1) no other tick bites were reported besides those due to the tested ticks, and 2) participants who reported that EM was treated with antibiotics for at least one week upon GP prescription. This left us with 14 strong EM cases. From 10 of these 14 cases, we received written informed consent to confirm the EM diagnosis through their GP; all these 10 EM diagnoses were confirmed by the GP (Table 1). This left us with 14 EM cases on which to base our analysis, of which 10 were also confirmed by their GP. The incubation time of these EM cases ranged from 7 to 44 days. We considered the outcomes of the other 11 EM cases as uncertain.

**Table 1:** Summary of epidemiological data from this study

**Outcome**	**Criteria**	**Frequency**	**Sensitivity**	**Specificity**
Self-reported EM	● Self-reporting of EM between 0 and 3 months after reporting a tick bite	25	n/a	n/a
Self-reported	● Same as self-reported EM	14	0% (0–22)	n/a
EM treated with antibiotics	● No other tick bites reported besides those due to the tested ticks			
	● Self-reporting of GP prescribed antibiotic treatment for EM			
GP-confirmed EM	● Same criteria as self-reported EM, treated with antibiotics	10	0% (0–28)	n/a
	● Written GP-confirmation of the self-reported EM diagnosis			
No EM	● No reporting of EM between t = 0 and t = 3 months after a tick bite	45	n/a	100% (92–100)
Unknown	● Lost to follow-up after a tick bite	52	n/a	n/a

Ticks from 122 individuals tested negative with the the Careplus Tick-test. The 25 self-reported EM cases were critically evaluated: Only those with confirmation by their GP, were used to calculate the sensitivity of the self-test. Out of 14 self-reported EM cases treated with antibiotics, we received ten written informed consents allowing us to contact the GP for confirmation of the EM diagnosis. These ten EM cases were subsequently confirmed by their GPs.

The Careplus Tick-test was performed on all 127 ticks of the 122 individuals according to the manufacturer’s protocol except for a minor modification: the wooden stick was replaced by a plastic pestle in order to improve the homogenization of the tick. For the five individuals with two ticks, three times the two ticks were tested with one test, and twice the two ticks were tested separately. The self-test yielded negative results, regardless of whether the ticks were obtained from persons without EM-formation (n = 45), with EM-formation (n = 14), with uncertain EM-formation (n = 11) and with unknown outcome (n = 52). The Careplus Tick-test became positive when about 5.10^5^ spirochetes of a *B. burgdorferi* senso stricto culture (B31-strain) were applied on the membrane. Remnants of the tick lysates from the 14 persons with EM were subjected to DNA extraction and a duplex QPCR to test whether *B. burgdorferi* sensu lato was present [12]. *Borrelia* DNA was detected in samples of 9 of the 14 self-reported EM-cases (64%) and 6 out of 10 (60%) of the GP-confirmed EM-cases, indicating the presence of *B. burgdorferi* sensu lato spirochetes in the ticks of *at least* 64% and 60% of these cases respectively: The DNA detection was performed under suboptimal conditions, as less than 5% of the tick lysate could be used, whereas normally the whole tick lysate is used. Using the same QPCR in other ticks enrolled through the national web-based survey, 20% (444 out of 2213) of the ticks from persons without EM-formation tested positive.

All ticks from the 122 individuals in this study tested negative in the Careplus Tick-test. Table 1 shows the sensitivity and specificity for the prediction of EM formation of the Careplus Tick-test. The sensitivity was estimated to be 0% (95% CI: 0-22%) based upon the ticks of 14 persons who had been bitten and self-reported formation of EM and subsequent GP-prescribed antibiotic treatment [13]. If only the 10 GP-confirmed EMs were included, the sensitivity was also estimated at 0%, but with a higher 95% upper boundary (28%). The specificity was estimated to be 100% (95% CI: 92-100%), based upon the 45 individuals without EM. This self-test is not suitable for reducing the NNT in post-exposure prophylaxis as it missed all the obvious early Lyme borreliosis cases.

## Competing interests

No conflict of interests to declare.

## Authors’ contributions

HS, WP, CW and AV designed the study. MH, WP, AV, CW collected ticks, epidemiological data and provided to participants. MH, CW and JC performed the statistical and epidemiological analysis. AdL, MF set up and performed laboratory tests, and interpreted these results. HS, CW and WP interpreted the data. HS wrote the manuscript and all contributed to the final version. All authors read and approved the final version of the manuscript.
